# Management performance in public oral health actions and services: a cross-sectional study in the Serra Catarinense region, Brazil, 2022

**DOI:** 10.1590/S2237-96222025v34e20240184.en

**Published:** 2025-06-16

**Authors:** Priscila Nunes, Rafael Gomes Ditterich, Claudia Flemming Colussi, Heloisa Godoi, Ana Lúcia Schaefer Ferreira de Mello

**Affiliations:** 1 Universidade Federal de Santa Catarina, Programa de Pós-graduação em Odontologia, Florianópolis, SC, Brazil; 2 Universidade Federal do Paraná, Setor de Ciências da Saúde, Departamento de Saúde Coletiva, Curitiba, PR, Brazil; 3 Universidade Federal de Santa Catarina, Programa de Pós-graduação em Saúde Coletiva, Florianópolis, SC, Brazil

**Keywords:** Oral Health, Health Management, Health Policies, Health Services, Health Planning, Salud Bucal, Gestión en Salud, Políticas de Salud, Servicios de Salud, Planificación en Salud

## Abstract

**Objective:**

To evaluate municipal health service management performance regarding public oral health actions and services.

**Methods:**

Cross-sectional study conducted in the 18 municipalities of Serra Catarinense region, Brazil, in 2022, with health secretaries, primary care and oral health coordinators; performance was self-assessed considering three dimensions (planning, monitoring and evaluation, and specific actions) and classified as poor (<2), medium (2-3) and good (>3); analyses were carried out according to management functions, using Kruskal-Wallis and Scheffé means comparison tests and analysis of variance, taking a 5% significance level.

**Results:**

Overall performance was evaluated as medium, with higher averages for planning (2.51; 95% confidence interval [CI] 2.32; 2.69); the specific actions dimension had the poorest performance (2.28 95%CI 2.08; 2.48). Health secretaries had higher performance averages when compared to coordinators of other functions (3.22; p-valor 0.007).

**Conclusion:**

Health service manager performance was considered to be medium, with differences depending on the managers’ role. Specific oral health actions had the poorest performance.

## Resumen

**Objetivo:**

Evaluar el desempeño de la gestión en las acciones y servicios públicos de salud bucal de los gestores municipales.

**Métodos:**

Estudio transversal realizado en los 18 municipios de la Sierra Catarinense, Brasil, en 2022, con secretarias de salud, coordinadores de atención primaria y salud bucal; el desempeño se autoevaluó considerando tres dimensiones (planificación, monitoreo y evaluación, y acciones específicas) y se clasificó como malo (<2), moderado (2-3) y bueno (>3); Los análisis se realizaron según funciones de gestión, utilizando las pruebas de comparación de promedios de Kruskal-Wallis y Scheffé y análisis de varianza, adoptando un nivel de significancia del 5%.

**Resultados:**

El desempeño general se evaluó como moderado, con promedios más altos en planificación (2,51; intervalo de confianza [IC] del 95%2,32; 2,69); la dimensión acciones específicas tuvo el peor desempeño (2,28 IC95%2,08; 2,48). Los secretarios de salud tuvieron promedios de desempeño más altos en comparación con otras funciones (3,22; p-valor 0,007).

**Conclusión:**

El desempeño de los gestores fue considerado moderado, con diferencias según el rol de gestión. Las acciones específicas de salud bucal tuvieron peores resultados.

**Ethical aspects**

This research respected ethical principles, having obtained the following approval data: :

Research Ethics Committee : Universidade Federal de Santa Catarina

Opinion Number : 5.180.033

Approval Date: 20/12/2021

Certificate of Submission for Ethical Appraisal : 54379821.3.0000.0121

Informed Consent Form : Obtained from all participants prior to data collection.

## Introduction 

Performance involves continuous analysis of indicators of the processes and effectiveness of services provided, being a fundamental parameter for the governance of health systems (1). In public service management, performance is assessed by the ability to establish and achieve goals, monitor access to services and the results obtained, that is, it concerns planning, monitoring and evaluation (2). These dimensions, when well evaluated, contribute to the effectiveness of public health actions and services, especially when associated with integrated action by managers and a close relationship between health service management and health care (3-6). 

The National Oral Health Policy is the basis for the management of oral health services, aiming to expand availability and access to services in the Brazilian National Health System (*Sistema Único de Saúde* - SUS). Implementation of the Policy requires strategic and efficient public management to promote oral health among the population (7). In this context, management performance is directly linked to the organization, coordination, monitoring and implementation of concrete actions (8). Management capacity, integration of management levels and efficient use of resources impact the resolutive capacity of dental services and service user satisfaction (3,9). Communication, engagement of health professionals and the promotion of a permanent learning environment are important elements for the performance of public health managers (2).

The practices of oral health professionals are influenced by their managers (8,10,11). The profile of managers, their degree of technical knowledge, the manner in which they are employed as managers and the way they manage and integrate the oral health network are sensitive points that can impact the performance of oral health management (12-14). It can therefore be seen that the oral health management process in the public sector and, consequently, its performance, continue to present significant gaps, especially in areas such as coordination of the care network, evaluation and monitoring of indicators, communication capacity and community participation, in addition to ensuring the necessary infrastructure and resources (15,16). The lack of current evidence on the performance of oral health management, specifically from the point of view of municipal health service managers, makes further investigations necessary. Given this context, the objective of this study was to evaluate municipal health service management performance regarding public oral health actions and services.

## Methods 

### Design and background

This is a cross-sectional study conducted between May and July 2022, with health service managers from 18 municipalities in the Região Serrana, located in the central part of the state of Santa Catarina, in southern Brazil. 

### Participants

Managers in positions of oral health coordinators, primary care coordinators and municipal health secretaries were eligible to participate. There was no sample size calculation.

### Variables

The study was conducted considering sociodemographic and oral health management variables, namely: sex (female, male), age (in years: 20-40, ≥41), higher education, time since graduation in higher education (in years: <6 , ≥6), time working as a manager (in years: <5, ≥5), type of employment (permanent, temporary), weekly workload (in hours: 20-30, 40), postgraduate degree in the technical area (yes, no), postgraduate degree in management (yes, no). Three dimensions were considered as variables in relation to management of public oral health actions and services, namely: planning, monitoring and evaluation, and specific actions. 

### Data source and measurement

The study was carried out through remote consultation. The invitation to participants was sent electronically, containing the research objectives and methodology, as well as a link to access the data collection instrument (questionnaire) and the free and informed consent form. The questionnaire was hosted on the Google Forms platform, with questions previously validated regarding the clarity and objective of the study, by a group of six professionals with expertise in the area of oral health management. The questionnaire was composed of 25 questions prepared through adaptation of indicators used to evaluate oral health management in primary health care (PHC) (17) and factors that interfere with compliance with the National Oral Health Policy in PHC (18). 

Each question gave rise to its corresponding variable and, as detailed in Table 1, the questions were organized into three dimensions: planning (9 questions), monitoring and evaluation (10 questions) and specific oral health actions (6 questions). A score was given for each planning dimension answer option: No/Unable to answer=0 and Yes=4, and for Likert scale answers to the other dimensions: never=0, rarely=1, sometimes=2, often=3 and, very often=4. A value judgment was made for each variable, dimension and total set of variables, regarding performance: poor (average values less than 2), medium (average values between 2 and 3) and good (values above 3). 

**Table 1:** Description of the study dimensions and variables

Dimensions	Variables	Answer score range
Planning		
1. Do you participate/have you participated in the preparation of municipal health plans	Participation in the preparation of municipal plans	Unable to answer (0) No (0) Yes (4)
2. Do you participate/have you participated in meetings with other health managers in your municipality??	Participation in meetings with other managers	
3. Do you (and/or your team) plan oral health actions and services as part of municipal management?	Planning of oral health actions and services	
4. Does the planning of oral health actions in the municipality take into account the available local epidemiological information?	Use of local epidemiological information	
5. Do you keep up with and participate in deliberations regarding oral health at the Municipal Health Conference?	Keeping up with municipal deliberations	Never (0) Rarely (1) Sometimes (2) Often (3) Very often (4)
6. Do you participate in meetings of the Municipal Health Council that discuss topics related to oral health?	Participation in municipal health councils	
7. Do you request and/or guarantee investments in oral health as part of the total amount invested in health in the municipality?	Guarantee of investment in oral health	
8. Do you know and keep up with the resources made available by the Ministry of Health and the State Health Department?	Keeping up with allocation of federal resources	
9. In the management of oral health in your municipality, are Ministry of Health indicators and/or locally defined indicators used in the action planning process?	Use of indicators in planning oral health actions	
**Monitoring and evaluation**		
1. How often does oral health management evaluate the work process of its professionals in primary care?	Evaluation of the work process in primary care	Never (0) Rarely (1) Sometimes (2) Often (3) Very often (4)
2. How often does management evaluate the results of the municipality’s oral health actions and services?	Evaluation of the results of oral health actions and services	
3. How often does management monitor the municipality’s oral health indicators?	Oral health indicator monitoring	
4. Does management evaluate the percentage that oral health accounts for in the Family Health Strategy?	Oral health teams expressed as a proportion of the Family Health Strategy	
5. Does management monitor health centers where service has been interrupted for more than two consecutive days, due to lack of oral health services professionals, supplies or equipment?	Oral health service interruption monitoring	
6. Does management monitor percentage of coverage of oral health services in the municipality?	Oral health service coverage monitoring	
7. Does management monitor the proportion of the number of consulting rooms in relation to the total population?	Number of oral health consulting rooms monitored	
8. Does management analyze the ratio between dental assistants and dental surgeons in primary care?	Analysis of the oral health staff ratio	
9. How often does lack of supplies hinder adequate oral health work process in primary care?	Frequency of lack of oral health supplies	
10. How often does lack of human resources hinder adequate oral health work process in primary care?	Frequency of lack of human resources in oral health services	
**Specific actions**		
1. Does management participate in ensuring food control in municipal school canteens?	Food control in school canteens	Never (0) Rarely (1) Sometimes (2) Often (3) Very often (4)
2. Does management ensure the existence of systematic collection of water fluoridation indicators and monitor them?	Surveillance of fluoride content in the water supply	
3. Does management participate in ensuring tooth brushing after snacks in public schools/daycare centers?	Tooth brushing ensured in schools	
4. Does management ensure educational and/or informative material about oral health?	Oral health educational material ensured	
5. Does management establish the number of dentist hours/population?	Number of dental surgeon hours/population established	
6. Does management ensure operational referral to oral health specialties?	Referral to dental specialties ensured	

### Bias 

The questionnaire was pre-tested by members of the research team to ensure it was understandable. The questionnaires were designed for remote use in order to minimize response and interpretation errors.

### Study size 

The study was conducted with all health service managers from the 18 municipalities, holding the following positions: health secretary, primary care coordinator and oral health coordinator. One manager was found to have two functions (health secretary and primary care coordinator), and one manager refused to participate in the study. 

### Statistical methods 

The analyses were performed using the Statistical Package for Social Sciences, version 1.6.2. Characterization of the participants was performed by means of descriptive analysis of absolute and relative frequencies. Data normality was tested using the Shapiro-Wilk test and homogeneity was tested using Levene’s test. The managers’ average scores and 95% confidence intervals (95%CI) were compared using the Kruskal-Wallis test for non-homogeneous samples without normal distribution. The Scheffé test was used to identify intergroup differences in the planning and the monitoring and evaluation dimensions. Homogeneous sample analysis of variance was used for the specific oral health action dimension. We used a 5% significance level.

## Results

A total of 52 managers participated in the study, predominantly women, and were 38.6 years old on average. Of these, 18 managers were responsible for oral health coordination, 17 were responsible for primary care coordination and 17 were health secretaries. All the oral health coordinators were dentists and all the primary care coordinators were nurses. The majority of health secretaries (n=9) had higher education in the field of health, had graduated six or more years ago (n=9), had up to five years of experience in health management (n=13), and had a temporary employment relationship (n=14). Half of the oral health coordinators (n=9) and the majority (n=12) of the primary care coordinators had a postgraduate degree in management. The majority (n=14) of health secretaries reported not having any postgraduate education in management (Table 2).

**Table 2:** Characterization of municipal oral health managers, Santa Catarina, 2022 (n=52)

Variables	Oral health	Primary care	Health secretary	Total
Sex				
Female	11	17	10	38
Male	07	-	07	14
**Age group** (years)				
20-40	13	09	04	26
≥41	05	08	13	26
**Higher education**				
Communication studies	-	-	01	01
Nursing	-	17	07	24
Pharmacology	-	-	01	01
Dentistry	18	-	-	18
Pedagogy	-	-	01	01
Management processes	-	-	01	01
Psychology	-	-	01	01
Information systems	-	-	01	01
Information technology	-	-	01	01
**Time since graduation** (years)				
<6	07	04	08	19
≥6	11	13	09	33
**Time as a manager** (years)				
<5	11	11	13	33
≥5	04	06	03	13
**Employment relationship**				
Permanent	10	10	03	23
Temporary	08	07	14	29
**Weekly working hours**				
20-30	03	01	01	05
40	12	17	14	42
**Postgraduate in the technical area**				
Yes	07	11	04	23
No	11	06	12	29
**Postgraduate in management**				
Yes	09	12	04	25
No	09	05	13	27

Better results were found for planning, while poor performance was found for specific oral health actions (Table 3).

**Table 3:** Distribution of average oral health management performance values, by dimension and management function, Santa Catarina, 2022 (n=52)

Dimension	Oral health	Primary care	Health secretary	Total
**Planning**				
Participation in the preparation of municipal plans	3.11	3.53	4.00	3.55
Participation in meetings with other managers	2.89	3.77	3.77	3.47
Planning of oral health actions and services	3.33	2.82	3.53	3.23
Use of local epidemiological information	2.00	2.35	3.29	2.55
Keeping up with municipal deliberations	1.83	2.47	2.82	2.38
Participation in municipal health councils	2.28	1.77	2.88	2.31
Guarantee of investment in oral health	2.00	2.65	2.94	2.53
Keeping up with allocation of federal resources	1.72	2.59	3.12	2.48
Use of indicators in planning oral health actions	2.06	1.94	2.65	2.22
**Total**	2.36	2.65	3.22	2.75
**Monitoring and evaluation**				
Evaluation of the work process in primary care	2.44	2.06	1.82	2.11
Evaluation of the results of oral health actions and services	2.72	2.71	3.24	2.89
Oral health indicator monitoring	2.94	2.82	3.06	2.94
Oral health teams expressed as a proportion of the Family Health Strategy	2.61	2.35	3.06	2.67
Oral health service interruption monitoring	2.50	2.47	3.12	2.70
Oral health service coverage monitoring	1.89	1.65	1.94	1.83
Number of oral health consulting rooms monitored	2.72	2.41	2.53	2.55
Analysis of the oral health staff ratio	1.89	2.18	2.41	2.16
Frequency of lack of oral health supplies	2.50	2.41	2.88	2.60
Frequency of lack of human resources in oral health services	2.44	2.41	2.65	2.50
**Total**	2.47	2.35	2.67	2.49
**Specific actions**				
Food control in school canteens	0.67	1.12	1.53	1.10
Surveillance of fluoride content in the water supply	1.56	1.82	2.41	1.92
Tooth brushing ensured in schools	2.61	2.18	2.18	2.32
Oral health educational material ensured	2.61	2.77	2.65	2.68
Number of dental surgeon hours/population established	3.06	3.00	2.53	2.87
Referral to dental specialties ensured	2.94	2.53	2.94	2.80
Total	2.24	2.24	2.37	2.28

In the planning dimension, the highest averages were found for “participation in the preparation of municipal health plans”, for both health secretaries and primary care coordinators; and, for the item “planning oral health actions and services”, for health secretaries and oral health coordinators. There were also high averages for “participation in meetings with other managers” for both primary care coordinators and secretaries, and for “use of epidemiological information” and “monitoring allocation of federal resources”, for health secretaries. The lowest averages were for “keeping up with oral health deliberations at municipal health conferences” and “keeping up with allocation of federal resources” among oral health coordinators. Primary care coordinators had the lowest averages in the item “use of indicators in planning oral health actions”.

In the monitoring and evaluation dimension, the items “evaluation of the results of actions and services”, “monitoring of oral health indicators”, “monitoring the proportion of oral health teams in the family health strategy” and “monitoring oral health service interruption” obtained the best averages for all groups. On the other hand, “monitoring oral health service coverage” obtained the lowest average among all management functions.

In the specific oral health action dimension, the best averages were for “establishment of the number of dentist surgeon hours/population” for oral health coordinators and primary care coordinators, and for “guarantee of referral to specialties” for all functions evaluated. On the other hand, low averages were found for “food control in school canteens” and “surveillance of fluoride content in water supply” among the three functions, and as such these were the items with the poorest evaluation.

Oral health management performance was medium (2.51; 95%CI 2.32; 2.69) (Table 4). The planning dimension planning had the highest average (2.74; 95%CI 2.49; 2.99). Health secretaries had higher average values in all three dimensions, so that their performance was considered good for the planning dimension (3.22 p-value 0.007) when compared to other management functions. In this dimension, statistically significant differences were found in the comparison between oral health coordinators and health secretaries (p-value 0.014), with a better result for the health secretaries.

**Table 4:** Average and confidence intervals (95%CI) of oral health management performance, according to the planning, monitoring and evaluation and specific actions dimensions per management function, Santa Catarina, 2022 (n=52)

Dimension	Oral health	Primary care	Health secretary	Total	p-valor
Planning	2.36 (1.84; 2.88)	2.65 (2.23; 3.08)	3.22 (2.95; 3.50)	2.74 (2.49; 2.99)	0.007^a^
Monitoring and evaluation	2.47 (1.97; 2.96)	2.35 (2.05; 2.64)	2.67 (2.47; 2.87)	2.49 (2.30; 2.69)	0.142^b^
Specific actions	2.24 (1.80; 2.68)	2.23 (1.90; 2.58)	2.37 (2.04; 2.71)	2.28 (2.08; 2.48)	0.829^c^
Total	2.35 (1.91; 2.80)	2.41 (2.12; 2.70)	2.75 (2.54; 2.97)	2.51 (2.32; 2.69)	0.238

^A^Kruskal-Wallis test for independent samples; ^b^Analysis of variance for homogeneous data; ^c^Scheffé test for intergroup differences.

No differences in performance were observed in oral health management, comparing the average values obtained by managers in the monitoring and evaluation dimension (p-value 0.142) and in the specific oral health actions dimension (p-value 0.829). The average performance of all managers was considered medium for the total average values (p-value 0.238) for these two dimensions.

## Discussion 

The performance of public management in oral health was evaluated as being medium, with the planning dimension obtaining better averages. A difference in performance was observed between oral health coordinators and health secretaries, with secretaries presenting better results in planning. The specific oral health actions dimension presented lower averages.

The differences in performance can be attributed to the different responsibilities of each role. Health secretaries, responsible for the macro management of the municipal health network, focus on strategic planning and resource allocation, thus explaining their better results in the planning dimension. Oral health coordinators, involved in implementing policies, face challenges in the practical implementation of actions, resulting in lower averages for specific actions. The coordinators of primary care and integration of health services also have difficulties in managing access and quality of dental care.

Evidence suggests that good practices and continuous management evaluation improve the effectiveness of oral health services (8-10,14-16). This study indicates the need for different approaches for each management function, with the aim of developing managerial skills in the municipal context, which can strengthen the planning, monitoring and provision of dental services, positively impacting the oral health of the population.

The results showed similarities with other national surveys involving SUS municipal managers, demonstrating a certain pattern, especially in relation to the characteristics of gender, education, training, experience and workload in municipal management (19,20). This study showed that oral health coordinators had graduated in the area of dentistry, and that primary care coordinators had graduated in the area of nursing. On the other hand, health secretaries showed diversity in their higher education, a fact that may be related to the political nature of their position (8). 

The way healthcare organization is managed directly influences the services offered and health outcomes. Dedication to work and management experience are associated with the success of health actions, especially in oral health (20). In addition, strengthening technical qualifications can integrate different levels of management and improve the overall performance of public management (22,23).

Two other points of discussion in this context involve managers’ time and the way in which they are employed. In this study, a workload of 40 hours per week predominated, and, for the most part, with a temporary employment relationship. Evidence shows that, in the health area, managers tend to accumulate functions, especially when employment relationships are unstable. In addition to the possibility of overburdening responsibilities, it can generate turnover in services, affecting management performance and, consequently, oral health care (20). Investing in manager training and providing adequate working conditions can assist oral health management performance.

Ability to plan, communicate, lead, master health policies and budget management are skills recognized as essential in public health management (2). In this study, in the planning dimension, higher averages stood out for health secretaries and primary care coordinators, especially in “participation in the preparation of municipal health plans” and “planning of oral health actions and services”. Good performance in this dimension demonstrates what science has been pointing out about the importance of managers in the formulation and implementation of oral health policies (23). Municipal management plays a fundamental role in reducing oral health inequalities by implementing planning tools that aim to guarantee universal and equitable access to oral health services (6). Management processes based on planning have the potential to mitigate regional disparities and disparities between groups regarding the population’s oral health conditions (24), their being essential in consolidated management instruments, such as municipal annual plans and schedules. The lower averages found for oral health coordinators for the “keeping up with oral health deliberations made at municipal health conferences” and “allocation of federal resources” indicators may indicate that there is little participation of managers in SUS decision-making bodies in order to ensure governance in the area (6).

In the monitoring and evaluation dimension, the health secretaries positively evaluated their performance, with emphasis on “oral health service interruption monitoring”. This may suggest commitment to the continuity and quality of services, as well as political concern about the negative impact of interrupting service availability (24). Higher averages were also found for “monitoring the proportion that oral health teams account for in the Family Health Strategy”, denoting commitment to the implementation and organization of these teams. Monitoring such metrics is essential for guaranteeing dental service coverage and continuously offering actions and services to the population (25).

The evaluation of the work process in PHC found lower averages for health secretaries, indicating a possible gap in this dimension (25). The results also showed that the “proportion of oral health teams”, as well as the “availability of human resources and supplies” were the items with the lowest performance averages among the three management functions. Management needs to include monitoring actions in order to guarantee adequate human resources and supplies, as well as the efficient organization of the work process, identifying specific points that may be compromising its performance (26). In general, evaluation of the work process in PHC can be a critical area, which requires attention and the development of specific strategies, as it demands the identification of indicators sensitive to the nuances of the teams’ work routine.

The specific oral health actions dimension was evaluated as having the lowest averages among all managers, with negative emphasis on actions of an intersectoral nature. Guaranteeing access to healthy food and adequate fluoride content in water supply constitutes health promotion and disease prevention measures, which need to be guaranteed by actions coordinated by management. Lack of food control in school canteens contributed to lower performance in this dimension, indicating possible weakness in promoting healthy eating practices in the school environment. From the perspective that schools are a privileged space for collective oral health activities (26), this result highlights the need to improve oral health intervention strategies in schools. 

One of the positive highlights in this dimension was that managers, in general, were able to establish the number of hours of dental surgeons in relation to the registered population and guarantee referral for access to dental specialties. It is in this integrated and responsive care context, through a solid care network, that robust clinical indicators of oral health resolutive capacity can be ensured (27,28).

The findings of this study have significant practical implications for oral health management and policy formulation to optimize service delivery. They are in line with the discussion in the literature about the need to improve the quality of management to guarantee a more efficient health system (8-10,14-16,29). This study explored how management performance in oral health can be improved through the adoption of work processes that value good management practices. Several strategies, such as quality programs, process improvements, financial incentives and performance monitoring measures, are essential for addressing the growing demand for healthcare services and ensuring the quality of care provided (23).

Studies on management performance assessment are relevant, but require critical analysis of conceptual and comparability limitations (1). Contrasting information can help management improve public health assessments, always considering the Brazilian context and best municipal practices (23,30). Despite portraying a local reality, the dimensions and indicators used can be applied to evaluate performance in other realities. Managers’ self-reflection on their performance is also important.

Limitations of this study include the use of the same parameters to evaluate the performance of the three management functions, given that each of them has specific responsibilities and different levels of decision-making. The instrument, despite being theoretically based, was developed specifically for this study and did not undergo external validation. The way in which the data was collected may also have influenced the results, since, when collected remotely, there is free interpretation by the participants, which may limit the understanding of the items. There may have been a tendency toward the halo effect, common in performance assessments that are limited to the analysis of one or a few factors, due to data collection being anonymized.

The aspects raised by this research indicate the need for more comprehensive investigations, including decision makers at state and federal levels. Identifying performance obstacles also points to opportunities to move towards health systems with improved provision of oral health actions and services. The conclusions reinforce the continuing need for research and interventions to improve oral health management and the quality of care offered to the population.

This study highlights the role of municipal management in qualifying oral health work in primary care. The results indicate the need to train managers and align professionals with management processes, creating an environment conducive to implementing oral health policies. Continuous development of managerial skills is essential to improve the planning, monitoring and evaluation of actions, address challenges and guarantee equitable, accessible and sustainable oral health services, in accordance with SUS guidelines. In this way it is possible to address current and future challenges in the field of oral health more efficiently, ensuring quality health care for the population.

## Data Availability

Not applicable.
