# Supplementary material for: Management performance in public oral health actions and services: a cross-sectional study in the Serra Catarinense region, Brazil, 2022
Source: Epidemiol Serv Saude. 2025 Jun 16;34:e20240184. doi: 10.1590/S2237-96222025v34e20240184.en (PMC12176441; doi:10.1590/S2237-96222025v34e20240184.en)
Supplement: Translation

# Translation: Desempenho da gestão nas ações e serviços públicos de saúde bucal: estudo transversal na região serrana de Santa Catarina, 2022

**DOI:** 10.1590/S2237-96222025v34e20240184.pt

**Authors:** Priscila Nunes, Rafael Gomes Ditterich, Claudia Flemming Colussi, Heloisa Godoi, Ana Lúcia Schaefer Ferreira de Mello

**Affiliations:** 1 Universidade Federal de Santa Catarina, Programa de Pós-graduação em Odontologia, Florianópolis, SC, Brasil; 2 Universidade Federal do Paraná, Setor de Ciências da Saúde, Departamento de Saúde Coletiva, Curitiba, PR, Brazil; 3 Universidade Federal de Santa Catarina, Programa de Pós-graduação em Saúde Coletiva, Florianópolis, SC, Brasil

**Keywords:** Saúde Bucal, Gestão em Saúde, Políticas de Saúde, Serviços de Saúde, Planejamento em Saúde

## Abstract

**Objetivo:**

Avaliar o desempenho da gestão nas ações e serviços públicos de saúde bucal de gestores municipais.

**Método:**

Estudo transversal conduzido nos 18 municípios da Serra Catarinense em 2022, com secretários de saúde, coordenadores de atenção primária e de saúde bucal; o desempenho foi autoavaliado considerando três dimensões (planejamento, monitoramento e avaliação, e ações específicas) e classificado considerando ruim (<2), moderado (2-3) e bom (>3); análises foram realizadas segundo as funções da gestão, por meio de testes de comparação de médias Kruskal-Wallis e de Scheffé e análise de variância, adotando e significância de 5%.

**Resultado:**

O desempenho geral foi avaliado como moderado, com maiores médias em planejamento (2,51; intervalo de confiança [IC] de 95% 2,32; 2,69); a dimensão ações específicas obteve o pior desempenho (2,28 IC95% 2,08; 2,48). Os secretários de saúde apresentaram maiores médias de desempenho quando comparados às demais funções (3,22; p-valor 0,007).

**Conclusão:**

O desempenho dos gestores foi considerado moderado, com diferenças segundo a função dos gestores. As ações específicas de saúde bucal apresentaram pior desempenho.

**Aspectos éticos**

A presente pesquisa respeitou os princípios éticos, obtendo os seguintes dados de aprovação::

Comitê de ética em pesquisa: Universidade Federal de Santa Catarina

Número do parecer: 5.180.033

Data de aprovação: 20/12/2021

Certificado de apresentação de apreciação ética: 54379821.3.0000.0121

Termo de consentimento: Obtido de todos os participantes antes da coleta dos dados.

## Introdução

O desempenho envolve a análise contínua dos processos e indicadores de eficácia dos serviços prestados, sendo um parâmetro fundamental para a governança dos sistemas de saúde (1). Na gestão pública, o desempenho é avaliado pela capacidade de estabelecer e alcançar metas, monitorar o acesso aos serviços e os resultados obtidos, ou seja, diz respeito ao planejamento, monitoramento e avaliação (2). Essas dimensões, quando bem avaliadas, contribuem para a efetividade das ações e serviços na área da saúde, em especial, quando associadas a uma atuação integrada dos gestores e a estreita relação da gestão com a atenção à saúde (3-6). 

A Política Nacional de Saúde Bucal fundamenta a gestão, visando ampliar a oferta e o acesso de serviços do Sistema Único de Saúde (SUS). Sua implementação requer gestão pública estratégica e eficiente para promover a saúde bucal da população (7). Nesse contexto, o desempenho da gestão está diretamente ligado à organização, coordenação, monitoramento e realização de ações concretas (8). A capacidade de gerenciamento, a integração dos níveis de gestão e o uso eficiente de recursos, impacta na resolutividade dos serviços odontológicos e na satisfação dos usuários (3,9). A comunicação, o engajamento dos profissionais de saúde e a promoção de um ambiente de aprendizado permanente, são elementos importantes para o desempenho de gestores na saúde pública (2).

As práticas dos profissionais de saúde bucal são influenciadas pela gestão (8,10,11). O perfil dos gestores, o grau de conhecimento técnico, a forma como são vinculados à gestão e a maneira como gerenciam e integram a rede de saúde bucal são pontos sensíveis e que podem impactar o desempenho da gestão da saúde bucal (12-14). Observa-se assim que, o processo de gestão da saúde bucal no setor público e, por consequência, o seu desempenho continuam apresentando lacunas significativas, sobretudo em áreas como coordenação da rede de atenção, avaliação e monitoramento de indicadores, capacidade de comunicação e participação comunitária, além da garantia de infraestrutura e recursos necessários (15,16). A ausência de evidências atuais sobre o desempenho da gestão em saúde bucal, especificamente, do ponto de vista dos gestores municipais, torna necessário novas investigações. Considerando o contexto, este estudo tem o objetivo de avaliar o desempenho da gestão nas ações e serviços públicos de saúde bucal de gestores municipais.

## Métodos

### Delineamento e contexto

Trata-se de estudo transversal realizado entre os meses de maio e julho de 2022, com gestores dos 18 municípios da região serrana, localizada no centro do estado de Santa Catarina, sul do Brasil. 

### Participantes

Foram elegíveis gestores que ocupavam funções de coordenadores de saúde bucal, coordenadores de atenção primária e secretários municipais de saúde. Não houve cálculo amostral.

### Variáveis

O estudo foi realizado considerando variáveis sociodemográficas e de gestão da saúde bucal, a saber: sexo (feminino, masculino), idade (em anos: 20-40, ≥41), formação superior, tempo de formação superior (em anos: <6, ≥6), tempo de atuação como gestor (em anos: <5, ≥5), tipo de vínculo (efetivo, temporário), carga semanal de trabalho (em horas: 20-30, 40), pós-graduação na área técnica (sim, não), pós-graduação na área da gestão (sim, não). Para a gestão das ações e serviços públicos de saúde bucal foram consideradas como variáveis três dimensões: planejamento, monitoramento e avaliação, e ações específicas. 

### Fonte de dados e mensuração 

O estudo foi realizado por meio de consulta remota. O convite aos participantes foi encaminhado por meio eletrônico, contendo os objetivos e a metodologia da pesquisa, além de um link de acesso ao instrumento de coleta de dados e ao termo de consentimento livre e esclarecido. O instrumento, do tipo questionário, foi elaborado na plataforma Google Forms, com questões validadas previamente quanto à clareza e objetivo do estudo, por um grupo de seis profissionais com expertise na área da gestão da saúde bucal. O instrumento foi composto por 25 questões elaboradas a partir de adaptação dos indicadores utilizados para avaliação da gestão da saúde bucal na atenção primária em saúde (APS) (17) e fatores que interferem no cumprimento da Política Nacional de Saúde Bucal na APS (18). 

Para cada opção de resposta foi atribuída uma pontuação correspondente: Não/Não sei=0 e Sim=4, e para as respostas da escala Likert: nunca=0, raramente=1, ocasionalmente=2, frequentemente=3 e, muito frequentemente=4. Cada questão deu origem à variável correspondente e, conforme detalhado na Tabela 1, essas questões foram organizadas em três dimensões: planejamento (9 questões), monitoramento e avaliação (10 questões) e ações específicas em saúde bucal (6 questões). Para cada variável, dimensão e conjunto total de variáveis, foi aplicado um juízo de valor sendo considerado desempenho: ruim (valores médios menores que 2), moderado (valores médios entre 2 e 3) e bom (valores acima de 3). 

**Tabela 1:** Descrição das dimensões e variáveis do estudo

Dimensões	Variáveis	Escala de resposta
Planejamento		
1. Você participa/participou da elaboração dos planos municipais de saúde?	Participação na elaboração dos planos municipais	Não sei (0) Não (0) Sim (4)
2. Você participa/participou de reuniões com os demais gestores da saúde do seu município?	Participação em reuniões com outros gestores	
3. Você (e/ou sua equipe) realiza o planejamento das ações e serviços da saúde bucal na gestão municipal?	Realização de planejamento de ações e serviços em saúde bucal	
4. O planejamento das ações da saúde bucal do município considera as informações epidemiológicas locais disponíveis?	Uso de informações epidemiológicas locais	
5. Acompanha e participa das deliberações referentes à saúde bucal na Conferência Municipal de Saúde?	Acompanhamento de deliberações municipais	Nunca (0) Raramente (1) Ocasionalmente (2) Frequentemente (3) Muito frequentemente (4)
6. Participa das reuniões do Conselho Municipal de Saúde que discutem temas afins à saúde bucal?	Participação nos conselhos municipais de saúde	
7. Pleiteia e/ou garante investimentos em saúde bucal do total investido na saúde no município?	Garantia de investimento em saúde bucal	
8. Conhece e acompanha os recursos disponibilizados pelo Ministério da Saúde e pela Secretaria do Estado?	Acompanhamento da alocação dos recursos federais	
9. Na gestão da saúde bucal do seu município são utilizados os indicadores do Ministério da Saúde e/ou indicadores definidos localmente no processo de planejamento das ações?	Uso de indicadores no planejamento das ações de saúde bucal	
**Monitoramento e avaliação**		
1. Com que frequência a gestão da saúde bucal avalia o processo de trabalho dos profissionais na atenção primária?	Avaliação do processo de trabalho na atenção primária	Nunca (0) Raramente (1) Ocasionalmente (2) Frequentemente (3) Muito frequentemente (4)
2. Com que frequência a gestão avalia os resultados das ações e serviços da saúde bucal do município?	Avaliação dos resultados das ações e serviços da saúde bucal	
3. Com que frequência a gestão monitora os indicadores da saúde bucal do município?	Monitoramento de indicadores de saúde bucal	
4. Avalia o percentual de saúde bucal na Estratégia Saúde da Família?	Proporção de equipes de saúde bucal na Estratégia Saúde da Família?	
5. Monitora as unidades de saúde em que houve interrupção do atendimento por mais de dois dias consecutivos, por falta de profissional dos serviços de saúde bucal, insumos ou equipamentos?	Monitoramento da paralisação dos serviços de saúde bucal	
6. Monitora o percentual de cobertura dos serviços da saúde bucal no município?	Monitoramento da cobertura dos serviços de saúde bucal	
7. Monitora a proporção do número de consultórios em relação à total população?	Monitoramento do número de consultórios de saúde bucal	
8. Analisa a proporção entre auxiliar e cirurgião-dentista na atenção primária?	Análise da proporção de equipe de saúde bucal	
9. Com que frequência a falta de insumos dificulta o adequado processo de trabalho da saúde bucal na atenção primária?	Frequência da falta de insumos de saúde bucal	
10. Com que frequência a falta de recurso humanos dificultando o adequado processo de trabalho da saúde bucal na atenção primária?	Frequência da falta de recursos humanos na saúde bucal	
**Ações específicas**		
1. Participa da garantia do controle de alimento nas cantinas das escolas municipais?	Controle de alimentos nas cantinas das escolas	Nunca (0) Raramente (1) Ocasionalmente (2) Frequentemente (3) Muito frequentemente (4)
2. Acompanha a existência de coleta sistemática e monitora indicadores de fluoretação de águas?	Vigilância do teor de flúor nas águas de abastecimento	
3. Participa da garantia da escovação dental após lanche nas escolas/creches públicas?	Garantia da escovação dental nas escolas	
4. Garante o material educativo e/ou informativo em saúde bucal?	Garantia de materiais educativos em saúde bucal	
5. Estabelece o número de horas cirurgião-dentista/população?	Estabelecimento do número de horas cirurgião-dentista/população	
6. Garante a referência operante para as especialidades em saúde bucal?	Garantia de referência para especialidades odontológicas	

### Viés 

O questionário foi testado previamente pelos membros da equipe da pesquisa, para assegurar a compreensão. Os questionários foram planejados para uso remoto, a fim de minimizar erros na resposta e em interpretações.

### Tamanho do estudo 

A pesquisa foi realizada com todos os gestores, dos 18 municípios, em três funções: secretário de saúde, coordenador da atenção primária e coordenador de saúde bucal. Observou-se que um gestor acumulava duas funções (secretária e coordenadora da atenção primária), e um gestor não aceitou participar do estudo. 

### Métodos estatísticos 

As análises foram realizadas no software Statistical Package for Social Sciences, versão 1.6.2. Para a caracterização dos participantes foi realizada análise descritiva das frequências absoluta e relativa. A normalidade dos dados foi investigada com o teste de Shapiro-Wilk e a homogeneidade pelo teste de Levene. Foram comparadas as médias e intervalo de confiança de 95% (IC95%) obtidas pelos gestores por meio do teste de Kruskal-Wallis para amostras sem distribuição normal e não homogêneas. Para identificar diferenças intergrupos, nas dimensões planejamento, monitoramento e avaliação, utilizou-se o teste de Scheffé. Para a dimensão ações específicas em saúde bucal foi aplicada análise de variância para amostras homogêneas. Adotou-se nível de significância de 5%.

## Resultados

Participaram do estudo 52 gestores, predominantemente mulheres, com média de idade de 38,6 anos. Destes, 18 gestores ocupavam a coordenação de saúde bucal, 17 a coordenação de atenção primária e 17 ocupavam a função de secretários de saúde. Em sua totalidade, os coordenadores de saúde bucal eram cirurgiões-dentistas e, os coordenadores de atenção primária, enfermeiros. A maioria dos secretários de saúde (n=9) tinha formação superior na área da saúde, havia concluído a formação há seis anos ou mais (n=9), com até cinco anos de experiência na gestão em saúde (n=13), com vínculo empregatício temporário (n=14). Metade dos coordenadores de saúde bucal (n=9) e a maioria (n=12) dos coordenadores de atenção primária, possuíam pós-graduação em gestão. A maioria (n=14) dos secretários de saúde relataram não ter nenhuma formação complementar em gestão (Tabela 2).

**Tabela 2:** Caracterização dos gestores da saúde bucal dos municípios, região serrana de Santa Catarina, 2022 (n=52)

Variáveis	Saúde bucal	Atenção primária	Secretário de saúde	Total
Sexo				
Feminino	11	17	10	38
Masculino	07	-	07	14
**Faixa etária** (anos)				
20-40	13	09	04	26
≥41	05	08	13	26
**Formação superior**				
Comunicação social	-	-	01	01
Enfermagem	-	17	07	24
Farmácia	-	-	01	01
Odontologia	18	-	-	18
Pedagogia	-	-	01	01
Processos gerenciais	-	-	01	01
Psicologia	-	-	01	01
Sistemas de informação	-	-	01	01
Tecnologia da informação	-	-	01	01
**Tempo de formação** (anos)				
<6	07	04	08	19
≥6	11	13	09	33
**Tempo na gestão** (anos)				
<5	11	11	13	33
≥5	04	06	03	13
**Vínculo de trabalho**				
Efetivo	10	10	03	23
Temporário	08	07	14	29
**Carga horária semanal**				
20-30	03	01	01	05
40	12	17	14	42
**Pós-graduação na área técnica**				
Sim	07	11	04	23
Não	11	06	12	29
**Pós-graduação na área de gestão**				
Sim	09	12	04	25
Não	09	05	13	27

Resultados melhores foram observados no planejamento e, desempenho ruim, na dimensão das ações específicas em saúde bucal (Tabela 3).

**Tabela 3:** Distribuição dos valores médios de desempenho da gestão em saúde bucal de acordo com as dimensões e função de gestão, região serrana de Santa Catarina, 2022 (n=52)

Dimensão	Saúde bucal	Atenção primária	Secretário de saúde	Total
**Planejamento**				
Participação na elaboração dos planos de saúde	3,11	3,53	4,00	3,55
Participação em reuniões com outros gestores	2,89	3,77	3,77	3,47
Realização de planejamento de ações e serviços	3,33	2,82	3,53	3,23
Uso de informações epidemiológicas locais	2,00	2,35	3,29	2,55
Acompanhamento de deliberações municipais	1,83	2,47	2,82	2,38
Participação no Conselho Municipal de Saúde	2,28	1,77	2,88	2,31
Garantia de investimento	2,00	2,65	2,94	2,53
Acompanhamento da alocação dos recursos	1,72	2,59	3,12	2,48
Uso de indicadores no planejamento das ações	2,06	1,94	2,65	2,22
**Total**	2,36	2,65	3,22	2,75
**Monitoramento e avaliação**				
Avaliação do processo de trabalho	2,44	2,06	1,82	2,11
Avaliação dos resultados das ações e serviços	2,72	2,71	3,24	2,89
Monitoramento de indicadores	2,94	2,82	3,06	2,94
Monitoramento da proporção de equipes	2,61	2,35	3,06	2,67
Monitoramento da paralisação dos serviços	2,50	2,47	3,12	2,70
Monitoramento da cobertura dos serviços	1,89	1,65	1,94	1,83
Monitoramento do número de consultórios	2,72	2,41	2,53	2,55
Proporção cirurgião-dentista e auxiliares	1,89	2,18	2,41	2,16
Frequência da falta de insumos	2,50	2,41	2,88	2,60
Frequência da falta de recursos humanos	2,44	2,41	2,65	2,50
**Total**	2,47	2,35	2,67	2,49
**Ações específicas**				
Controle de alimentos nas cantinas das escolas	0,67	1,12	1,53	1,10
Vigilância do flúor nas águas de abastecimento	1,56	1,82	2,41	1,92
Garantia da escovação dental nas escolas	2,61	2,18	2,18	2,32
Garantia de materiais educativos	2,61	2,77	2,65	2,68
Número de horas cirurgião-dentista/população	3,06	3,00	2,53	2,87
Garantia de referência para especialidades	2,94	2,53	2,94	2,80
Total	2,24	2,24	2,37	2,28

Na dimensão planejamento, as maiores médias foram observadas em “participação na elaboração dos planos municipais de saúde”, tanto entre secretários de saúde quanto entre coordenadores de atenção primária; e, no item “planejamento das ações e serviços de saúde bucal”, entre secretários de saúde e coordenadores de saúde bucal. Houve também médias altas em “participação em reuniões com outros gestores” entre coordenadores de atenção primária e secretários, e, em “utilização de informações epidemiológicas” e “acompanhamento da alocação de recursos federais”, entre secretários. As menores médias foram para “acompanhamento das deliberações da saúde bucal nas conferências municipais de saúde” e “alocação dos recursos federais” entre coordenadores de saúde bucal. Já os coordenadores de atenção primária apresentaram as menores médias no item “uso de indicadores no planejamento das ações de saúde bucal”.

Na dimensão monitoramento e avaliação, os itens “avaliação dos resultados das ações e serviços”, “monitoramento de indicadores da saúde bucal”, “monitoramento da proporção de equipes de saúde bucal na estratégia da saúde da família” e “monitoramento da paralisação do serviço de saúde bucal” obtiveram as melhores médias para todos os grupos. Por outro lado, “monitoramento da cobertura dos serviços de saúde bucal” obteve a menor média entre todos as funções de gestão.

Na dimensão ações específicas em saúde bucal, as melhores médias foram para “estabelecimento do número de horas cirurgião dentista/população” entre coordenadores de saúde bucal e coordenadores de atenção primária, e para “garantia de referência para especialidades” entre todas as funções avaliadas. Por outro lado, médias baixas foram observadas em “controle de alimentos nas cantinas das escolas” e “vigilância do teor de flúor nas águas de abastecimento” entre as três funções, sendo assim, os itens com pior avaliação.

O desempenho na gestão da saúde bucal foi moderado (2,51; IC95% 2,32; 2,69) (Tabela 4). A dimensão planejamento apresentou maior média (2,74; IC95% 2,49; 2,99). Secretários de saúde apresentaram valores médios superiores nas três dimensões, demonstrando um desempenho considerado bom na dimensão planejamento (3,22 p-valor 0,007) quando comparado às outras funções de gestão. Nesta dimensão, diferenças estatisticamente significativas foram encontradas na comparação entre coordenadores de saúde bucal e secretários de saúde (p-valor 0,014), com melhor resultado para o grupo dos secretários. 

**Tabela 4:** Média e intervalos de confiança (IC95%) do desempenho da gestão em saúde bucal, segundo as dimensões planejamento, monitoramento e avaliação e ações específicas por função de gestão, região serrana de Santa Catarina, 2022 (n=52)

Dimensão	Saúde bucal	Atenção primária	Secretário de saúde	Total	p-valor
Planejamento	2,36 (1,84; 2,88)	2,65 (2,23; 3,08)	3,22 (2,95; 3,50)	2,74 (2,49; 2,99)	0,007^a^
Monitoramento e avaliação	2,47 (1,97; 2,96)	2,35 (2,05; 2,64)	2,67 (2,47; 2,87)	2,49 (2,30; 2,69)	0,142^b^
Ações específicas	2,24 (1,80; 2,68)	2,23 (1,90; 2,58)	2,37 (2,04; 2,71)	2,28 (2,08; 2,48)	0,829^c^
Total	2,35 (1,91; 2,80)	2,41 (2,12; 2,70)	2,75 (2,54; 2,97)	2,51 (2,32; 2,69)	0,238

^A^Teste de Kruskal-Wallis para amostras independentes; ^b^Análise de variância para dados homogêneos; ^c^Teste de Scheffé para diferenças intergrupos.

Não foram observadas diferenças de desempenho na gestão da saúde bucal, comparando-se os valores médios obtidos pelos gestores nas dimensões monitoramento e avaliação (p-valor 0,142) e ações específicas de saúde bucal (p-valor 0,829), bem como nos valores médios totais (p-valor 0,238) para estas duas dimensões, o desempenho médio de todos os gestores foi considerado moderado.

## Discussão

O desempenho da gestão pública em saúde bucal foi avaliado como moderado, com a dimensão de planejamento obtendo melhores médias. Observou-se diferença no desempenho entre coordenadores de saúde bucal e secretários de saúde, com secretários apresentando melhores resultados no planejamento. A dimensão ações específicas em saúde bucal apresentou médias mais baixas.

As diferenças no desempenho podem ser atribuídas às responsabilidades distintas de cada função. Secretários de saúde, responsáveis pela gestão macro da rede municipal, concentram-se no planejamento estratégico e na alocação de recursos, explicando os melhores resultados na dimensão planejamento. Coordenadores de saúde bucal, envolvidos na execução das políticas, enfrentam desafios na implementação prática das ações, resultando em médias mais baixas em ações específicas. A coordenação da atenção primária e a integração dos serviços de saúde também apresentam dificuldades na gestão do acesso e da qualidade do atendimento odontológico.

Evidências sugerem que boas práticas e avaliação contínua da gestão melhoram a efetividade dos serviços de saúde bucal (8-10,14-16). Este estudo indica a necessidade de abordagens diferenciadas para cada função de gestão, visando desenvolver competências gerenciais no contexto municipal, o que pode fortalecer o planejamento, monitoramento e a oferta de serviços odontológicos, impactando positivamente a saúde bucal da população.

Os resultados mostraram semelhanças com outros levantamentos nacionais envolvendo gestores municipais do SUS, demonstrando certo padrão, especialmente em relação às características de sexo escolaridade, formação, experiência e carga de trabalho na gestão municipal (19,20). Foi evidenciado por este estudo que coordenadores de saúde bucal apresentaram formação na área de odontologia, assim como os de atenção primária na área de enfermagem. Por outro lado, os secretários de saúde apresentaram diversidade na formação superior, fato este que pode estar relacionado ao caráter político da função (8). 

A forma de gerenciar uma organização de saúde influencia diretamente nos serviços ofertados e nos desfechos em saúde. A dedicação ao trabalho e a experiência na gestão estão associadas ao sucesso das ações em saúde, especialmente em saúde bucal (20). Somado a isso, o reforço da qualificação técnica pode integrar diferentes níveis de gestão e melhorar o desempenho geral da gestão pública (22,23).

Outros dois pontos de discussão neste contexto envolvem o tempo e o regime de trabalho dos gestores. Neste estudo, predominou carga horária de 40 horas semanais, e, em sua maioria, com vínculo temporário. Evidências apontam que, na área da saúde, os gestores tendem acumular funções, em especial quando os vínculos são instáveis. Além da possibilidade da sobrecarga de responsabilidades, pode gerar rotatividade nos serviços, afetando o desempenho da gestão e, por consequência, a assistência em saúde bucal (20). Investir na capacitação dos gestores e proporcionar condições de trabalho adequadas podem auxiliar o desempenho da gestão em saúde bucal.

Capacidade de planejamento, comunicação, liderança, domínio das políticas de saúde e da gestão orçamentária são habilidades reconhecidas como essenciais na gestão em saúde pública (2). Neste estudo, na dimensão de planejamento, destacaram-se maiores médias para os secretários de saúde e coordenadores de atenção primária, especialmente na “participação da elaboração dos planos municipais de saúde” e “planejamento das ações e serviços de saúde bucal”. O desempenho considerado bom nessa dimensão, demonstra o que ciência vêm apontando sobre a importância dos gestores na formulação e implementação de políticas de saúde bucal (23). A gestão municipal desempenha um papel fundamental na redução das desigualdades em saúde bucal ao implementar ferramentas de planejamento que visam garantir acesso universal e equitativo aos serviços de saúde bucal (6). Os processos gerenciais baseados em planejamento têm o potencial de mitigar disparidades regionais e entre grupos nas condições de saúde bucal da população (24), sendo essenciais em instrumentos de gestão consolidados, como planos municipais e programações anuais. As médias mais baixas observadas nos indicadores de “acompanhamento das deliberações da saúde bucal nas conferências municipais de saúde” e “alocação dos recursos federais” para os coordenadores de saúde bucal, podem indicar que há pouca participação dos gestores em instâncias decisórias do SUS para garantir governança na área (6).

Na dimensão monitoramento e avaliação os secretários de saúde avaliaram positivamente o seu desempenho, com destaque para “monitorar a paralisação dos serviços de saúde bucal”. Isso pode sugerir um comprometimento com a continuidade e qualidade dos serviços, mas, também, uma preocupação política com o impacto negativo da interrupção da oferta (24). Médias mais altas foram observadas também no “monitoramento das proporções de equipes de saúde bucal na Estratégia Saúde da Família”, denotando compromisso com a implantação e organização dessas equipes. Acompanhar tais métricas é essencial para a garantir abrangência dos serviços odontológicos e ofertar continuamente ações e serviços à população (25).

A avaliação do processo de trabalho na APS teve médias mais baixas para os secretários de saúde, indicando possível lacuna nessa dimensão (25). Os resultados mostraram também que a “proporção de equipes de saúde bucal”, bem como a “disponibilidade de recursos humanos e insumos” foram os itens com menores médias de desempenho entre as três funções de gestão. Entende-se que a gestão deve incluir ações de monitoramento para garantir recursos humanos e insumos adequados, bem como, a organização eficiente do processo de trabalho, identificando pontos específicos que podem estar comprometendo o seu desempenho (26). De forma geral, a avaliação do processo de trabalho na APS pode ser área crítica, que requer atenção e desenvolvimento de estratégias específicas, uma vez que demanda identificação de indicadores sensíveis às nuances da rotina de trabalho das equipes.

A dimensão ações específicas em saúde bucal foi avaliada com as médias mais baixas entre todos os gestores, com destaque negativo para as ações de cunho intersetorial. A garantia de acesso à alimentação saudável e ao teor adequado de flúor nas águas de abastecimento constituem medidas de promoção da saúde e de prevenção de doenças, que devem ser afiançadas por ações coordenadas pela gestão. A falta de controle de alimentos nas cantinas das escolas contribuiu para um desempenho inferior nessa dimensão, indicando possível fragilidade na promoção de práticas alimentares saudáveis no ambiente escolar. Entendendo que a escola é um espaço privilegiado de atuação da saúde bucal coletiva (26), esse resultado ressalta a necessidade de aprimoramento das estratégias de intervenção na área da saúde bucal nas escolas. 

Um dos destaques positivos nesta dimensão foi que os gestores, de maneira geral, conseguiram estabelecer o número de horas dos cirurgiões-dentistas em relação à população adscrita e garantem a referência para acesso às especialidades odontológicas. É nesse contexto assistencial integrado e responsivo, por meio de sólida rede de atenção, que se pode assegurar indicadores clínicos robustos de resolutividade no campo da saúde bucal (27,28).

Os achados deste estudo têm implicações práticas significativas para a gestão em saúde bucal e a formulação de políticas para otimizar a prestação de serviços. Eles estão alinhados com a discussão na literatura sobre a necessidade de melhorar a qualidade da gestão para garantir um sistema de saúde mais eficiente (8-10,14-16,29). Este estudo explorou como o desempenho da gestão na saúde bucal pode ser aperfeiçoado por meio da adoção de processos de trabalho que valorizem boas práticas de gestão. Diversas estratégias, como programas de qualidade, aprimoramento de processos, incentivos financeiros e medidas de monitoramento de desempenho, são essenciais para enfrentar a crescente demanda por serviços de saúde e garantir a qualidade dos cuidados prestados (23).

Estudos sobre avaliação do desempenho da gestão são relevantes, mas requerem análise crítica das limitações conceituais e de comparabilidade (1). Contrastar informações pode ajudar a gestão a aprimorar avaliações em saúde pública, considerando sempre o contexto brasileiro e as melhores práticas municipais (23,30). Apesar de retratar uma realidade local, as dimensões e indicadores utilizados podem ser aplicados para avaliação de desempenho em outras realidades. A autorreflexão dos gestores sobre sua atuação também é importante.

Como limitações do estudo, podem ser citados a utilização dos mesmos parâmetros para avaliação de desempenho das três funções de gestão, assumindo que cada uma possui atribuições específicas e distintos níveis de tomada de decisão. O instrumento, apesar de ter sido fundamentado teoricamente, foi desenvolvido especificamente para este estudo e não passou por validação externa. A forma pela qual os dados foram coletados também pode ter influenciado os resultados, uma vez que, por meio de remoto, há a livre interpretação dos participantes, o que pode limitar a compreensão dos itens. Pode ter havido tendência ao efeito halo, comum em avaliações de desempenho que são limitadas a análise de um ou poucos fatores, em virtude da coleta ter sido anonimizada.

Os aspectos levantados por esta pesquisa indicam a necessidade de investigações mais abrangentes, incluindo tomadores de decisão em níveis estadual e federal. Identificar óbices no desempenho também aponta oportunidades para avançar em direção a sistemas de saúde com oferta qualificada de ações e serviços de saúde bucal. As conclusões reforçam a necessidade contínua de pesquisas e intervenções para melhorar a gestão em saúde bucal e a qualidade dos cuidados oferecidos à população.

Este estudo destaca o papel da gestão municipal na qualificação do trabalho em saúde bucal na atenção primária. Os resultados indicam a necessidade de capacitar gestores e alinhar profissionais aos processos gerenciais, criando um ambiente propício para implementar políticas de saúde bucal. O desenvolvimento contínuo das competências gerenciais é essencial para melhorar o planejamento, monitoramento e avaliação das ações, enfrentar desafios e garantir serviços de saúde bucal equitativos, acessíveis e sustentáveis, em conformidade com as diretrizes do SUS. Pode-se, assim, enfrentar de maneira mais eficiente os desafios presentes e futuros no campo da saúde bucal, garantindo atenção à saúde de qualidade à população.

## Data Availability

Não se aplica.
